# Correlation of atorvastatin with subjective memory deficits: a study from the NHANES and FAERS databases

**DOI:** 10.3389/fneur.2025.1526959

**Published:** 2025-03-10

**Authors:** Hao Zhang, Hua Huang, Panli Zhao

**Affiliations:** Department of Pharmacy, Chengdu Seventh People’s Hospital (Affiliated Tumor Hospital of Chengdu Medical College), Chengdu, China

**Keywords:** atorvastatin, memory, NHANES, FAERS, cognitive function

## Abstract

**Background:**

Post-marketing regulatory data suggest a potential association between atorvastatin use and memory protection; however, findings from observational studies have been inconsistent and remain a subject of controversy.

**Objective:**

This study aims to investigate the correlation between atorvastatin exposure and subjective memory deficits, with the objective of providing more precise safety and efficacy information for its clinical use.

**Methods:**

We utilized two primary data sources: the National Health and Nutrition Examination Survey (NHANES) covering the years 2001 to 2018, and the Food and Drug Administration Adverse Event Reporting System (FAERS) spanning 2011 to 2018. We systematically analyzed the correlation between atorvastatin exposure and memory function using a range of statistical methods, including descriptive statistics, multivariate logistic regression, and receiver operating characteristic (ROC) curves.

**Results:**

In the analysis of the NHANES database, multivariate logistic regression modeling, after controlling for various factors such as demographic characteristics and lifestyle, revealed a significant association between atorvastatin use and a reduced risk of memory loss (OR = 0.47; 95% CI, 0.15–0.79; *p* = 0.004). This suggests that atorvastatin may offer a protective effect on memory. Conversely, our analysis of the FAERS database identified 15,277 reports of adverse reactions associated with atorvastatin, of which 401 were related to psychiatric adverse events, including memory loss. This finding indicates that while atorvastatin may not generally increase the risk of memory loss, some patients may still experience these side effects.

**Conclusion:**

This study integrated data from NHANES and FAERS to provide a comprehensive analysis of the relationship between atorvastatin and memory function. On one hand, the NHANES findings support the potential benefits of atorvastatin in reducing the risk of memory loss. On the other hand, the FAERS data highlight specific cognitive side effects associated with the drug. Consequently, clinicians and patients should carefully consider both the potential benefits and risks of atorvastatin, taking into account individual patient differences and implementing appropriate monitoring strategies.

## 1 Introduction

Alzheimer’s disease (AD) is a prevalent neurodegenerative disorder primarily affecting the elderly, characterized by a progressive decline in cognitive abilities (1). Patients with AD experience significant memory loss, disorganized thinking, and behavioral changes. As the disease advances, individuals not only lose their independence but may also become entirely dependent on others for care (2, 3). Cognitive functioning encompasses several domains, including memory, attention, language, decision-making, and spatial perception, which are crucial for daily life. Memory, a fundamental aspect of cognitive functioning, involves the acquisition, storage, and retrieval of information, serving as the basis for recalling past experiences and knowledge (4). In the early stages of Alzheimer’s disease, patients often exhibit notable deficits in short-term memory, with long-term memory and other cognitive functions progressively deteriorating as the disease progresses (5, 6). Given the significant impact of Alzheimer’s disease on patients’ quality of life, research into methods to protect or delay cognitive decline, particularly memory loss, remains a critical focus of contemporary medical research.

Atorvastatin, a statin drug approved for marketing in 2003, is primarily used to treat hypercholesterolemia and prevent cardiovascular disease (7). It works by reducing cholesterol synthesis through the inhibition of hydroxymethylglutaryl coenzyme A reductase (HMG-CoA reductase) in hepatocytes. Additionally, atorvastatin enhances the hepatic clearance of low-density lipoprotein cholesterol (LDL-C) from the bloodstream by upregulating low-density lipoprotein receptors (LDL-receptors), thereby effectively lowering blood cholesterol levels (8–10). Since its introduction, atorvastatin’s applications have expanded, revealing potential therapeutic effects in various areas. It has been shown to reduce the risk of acute exacerbations in chronic obstructive pulmonary disease (COPD) patients through its anti-inflammatory and antioxidant properties (11). Moreover, atorvastatin demonstrates effectiveness in preventing cardiovascular events in individuals with chronic kidney disease (12) and offers potential benefits for patients with non-alcoholic fatty liver disease (NAFLD) by improving liver function and slowing disease progression (13). Recent studies suggest atorvastatin may also reduce mortality in certain cancers, including breast cancer (14) and colorectal cancer (15). In the neurological domain, atorvastatin exhibits complex effects on memory and cognitive function. Some studies indicate cognitive protective effects, particularly in the elderly and patients with cardiovascular disease, by slowing age-related cognitive decline, improving vascular function, and reducing brain inflammation and oxidative stress through its anti-inflammatory and antioxidant properties. Clinical research reports improvements in memory retention with long-term atorvastatin use, which is relevant for managing cardiovascular disease (16–20). However, atorvastatin’s impact on cognitive function is not uniformly positive. Some studies have linked long-term or high-dose use to cognitive decline, including memory loss and poor concentration (21–24). Although usually mild and reversible, these side effects may affect patients’ quality of life and treatment adherence. Although atorvastatin is highly effective in lowering cholesterol and preventing cardiovascular disease, prolonged use has been associated with several side effects. In addition to common issues like muscle pain (25) and arthralgia (26), atorvastatin may cause more serious problems, such as rhabdomyolysis (27, 28), hepatic dysfunction (29), new-onset diabetes mellitus (30), cognitive decline and memory problems (31–33), and, rarely, neuropathy (34). These side effects underscore the importance of risk assessment and close monitoring of patient response during atorvastatin therapy.

The aim of this study was to systematically evaluate the relationship between atorvastatin use and memory by analyzing the NHANES and FAERS databases. Additionally, the study sought to explore the potential effects of atorvastatin on subjective memory across different populations, providing a scientific basis and empirical support for clinical decision-making and medication management.

## 2 Materials and methods

Data Sources The National Health and Nutrition Examination Survey (NHANES) is a nationally representative survey conducted by the National Center for Health Statistics (NCHS). Participants are selected using a multi-stage, stratified probability sampling strategy. Data are collected through comprehensive household interviews, physical examinations at mobile examination centers (MECs), and blood sample analyses. The study protocol was approved by the Research Ethics Review Board of the National Center for Health Statistics. For this study, NHANES data from 2001 to 2018 were utilized to ensure consistency in covariate definitions, and all detailed information was sourced from the official NHANES website (35). OpenVigil 2.1^1^ is a widely used online tool for pharmacovigilance and data mining (36). We searched this database from January 1, 2011, to December 31, 2018, using the target drug name “Atorvastatin” to obtain data on primary suspect adverse drug reactions related to atorvastatin.

Exposure Factors We assessed atorvastatin exposure based on participants’ responses to prescription drug use. Specifically, participants were asked, “Have you taken or used any prescription drugs in the past month?” Those who responded “no” were classified as the control group, while individuals who indicated they had used atorvastatin were classified as the exposed group. Additionally, the prescription drug questionnaire collected information on the duration of medication use through the question: “How long have you been using or taking (product name)”?

Memory Status Participants in the NHANES database were evaluated for physical functioning during the Mobile Examination Center (MEC) assessment. In the “Experiencing Confusion/Memory” module, respondents were asked, “Are you limited because you have difficulty remembering or are often confused?” A response of “no” indicates that subjective memory is not significantly affected, while a response of “yes” suggests that subjective memory deficits have a considerable impact on daily life.

Covariates A range of potential covariates were examined in this study, including age, gender, ethnicity, education, body mass index (BMI), smoking status, caffeine intake, sugar intake, alcohol consumption, diabetes mellitus, hypertension, and physical activity. Age was treated as a continuous variable. Participants self-reported their race and were classified into five groups: Mexican American, other Hispanic, non-Hispanic white, non-Hispanic black, and other race. Educational attainment was categorized as high school graduate or less, some college, and college graduate or more. Smoking status was divided into two categories: never smokers (fewer than 100 cigarettes in their lifetime) and smokers (100 or more cigarettes). Physical activity was assessed based on the question, “In a typical week, do you engage in any moderate-intensity exercise, fitness, or recreational activities that result in a slight increase in breathing or heart rate, such as brisk walking, bicycling, swimming, or volleyball, and that last for at least 10 min each time?” Body mass index (BMI) was calculated as weight in kilograms divided by height in meters squared (kg/m^2^). Diabetes mellitus and hypertension were determined based on participants’ self-reports of whether they had been previously informed by a physician of the condition (37).

Statistical analysis Because NHANES utilizes a complex multi-stage stratified probability survey design, the statistical analysis for this study combined sample weighting, clustering, and stratification, with continuous variables expressed as means (standard deviation SD) and categorical variables expressed as counts (percentages). Logistic regression was used to estimate odds ratios (OR) and 95% confidence intervals (95% CI) for the association between atorvastatin use and memory function. Model 1 was unadjusted. Model 2 was based on model 1, adjusted for age, sex, race, and education level. Model 3 was fully adjusted, accounting for all variables in Model 2, in addition to physical activity, diabetes, hypertension, caffeine intake, alcohol consumption, and sugar intake. Interaction and subgroup analyses were also conducted using logistic regression models stratified by age group, gender, BMI, smoking status, hypertension, physical activity, and diabetes. Sensitivity analyses were performed to assess the robustness of the findings. First, E-Values were calculated after performing logistic regression on Model 3. Additionally, diagnostic performance was assessed using receiver operating characteristic (ROC) curves, and the area under the curve (AUC) was calculated to evaluate predictive accuracy (38). Furthermore, adverse events related to memory deficits were mined from the FAERS database to explore the real-world safety profile of atorvastatin. For FAERS data analysis, the reporting odds ratio (ROR) method was combined with the Bayesian Confidence Propagation Neural Network (BCPNN) method for signal detection (39), and the specific formulas are shown in Tables 1, 2. Each Preferred Term (PT) was screened against the thresholds outlined in Table 2. Both algorithms needed to meet the judgmental criteria before an adverse drug event (ADE) signal was generated. The generation of ADE signals indicates a statistical association with the drug, where a stronger signal implies a stronger correlation. All statistical analyses were conducted using R software version 4.4.0 and Excel, with statistical significance defined as *p* < 0.05.

**Table 1:** Fourfold table for calculation.

	Topotecan	Non-topotecan
Target AEs	a	c
Non-target AEs	b	d
N = a + b + c + d		

**Table 2:** Formulas and thresholds of ROR and BCPNN.

Method	Formula	Threshold
ROR	$ROR=\frac{a/c}{b/d}$ 95%CI=e^(lnROR±1.961/a+1/b+1/c+1/d)	a ≥ 3 and 95% CI (lower limit) > 1
BCPNN	$IC=\log_{2}\frac{a\left(a+b+c+d\right)}{\left(a+b\right)\left(a+c\right)}$ $\gamma =\gamma_{ij}\frac{\left(N+\alpha \right)\left(N+\beta \right)}{\left(a+b+\alpha_{i}\right)\left(a+c+\beta_{j}\right)}$ $E\left(IC\right)=\log_{2}\frac{\left(a+\gamma_{ij}\right)\left(N+\alpha \right)\left(N+\beta \right)}{\left(N+\gamma \right)\left(a+b+\alpha_{i}\right)\left(a+c+\beta_{j}\right)}$ $V\left(IC\right)={\left(\frac{1}{\ln2}\right)}^{2}\left(\frac{N-\alpha +\gamma -\gamma_{ij}}{\left(\alpha +\gamma_{ij}\right)\left(1+N+\gamma \right)}+\frac{N-a-b+\alpha -\alpha_{i}}{\left(a+b+\alpha_{i}\right)\left(1+N+\alpha \right)}+\frac{N-a-c+\beta -\beta_{j}}{\left(a+c+\beta_{j}\right)\left(1+N+\beta \right)}\right)$ $SD=\sqrt{V\left(IC\right)}$ $IC025=E\left(IC\right)-2SD$	IC025 > 0

## 3 Results

**Figure 1:**
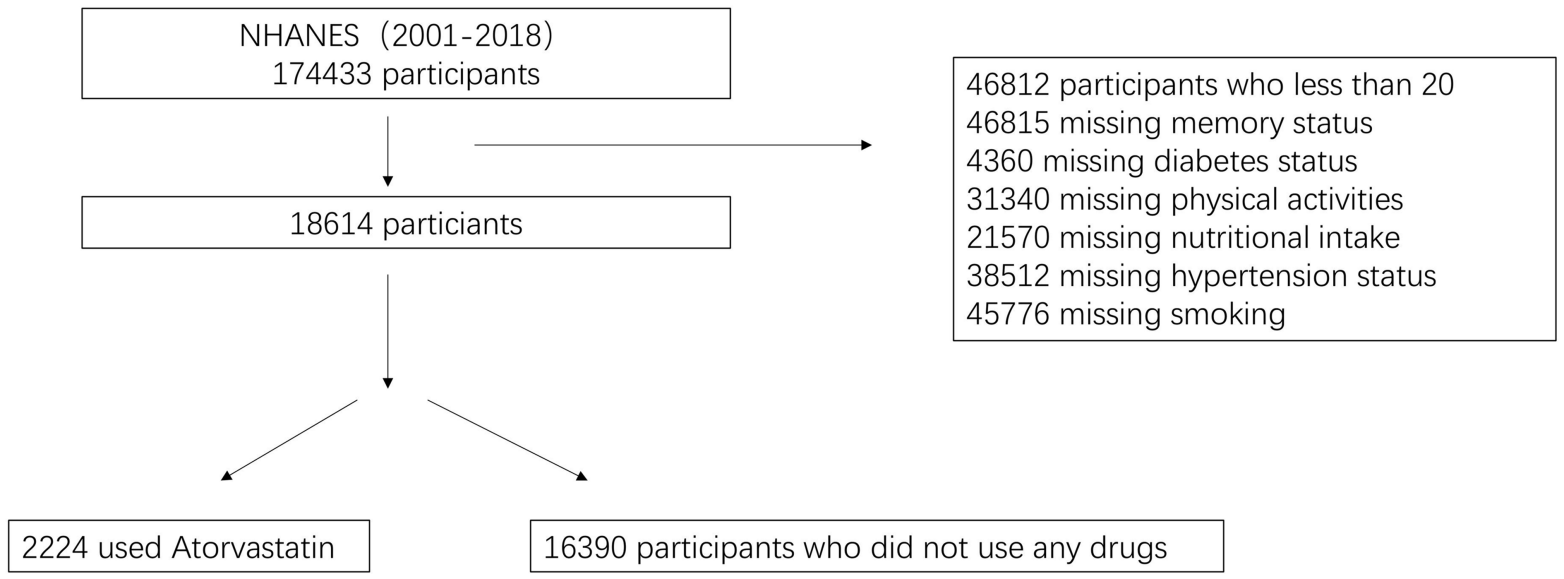
Flowchart of the sample selection from NHANES.

Characteristics of participants From the NHANES data spanning 2001 to 2018, a total of 18,614 subjects were included in this study following a rigorous screening process. Exclusions were made for individuals younger than 20 years of age, those with missing memory status, incomplete medication use data, missing information on nutritional intake, unknown status of hypertension and diabetes, unknown smoking status, and insufficient data on physical activity. The remaining subjects were categorized into two groups based on atorvastatin use: the atorvastatin group (*n* = 2,224) and the non-atorvastatin group (*n* = 16,390). Among these, 797 subjects reported memory deficits, while 17,817 did not. The mean age of the subjects was 42 years, with a gender distribution of 58% male and 42% female. Notably, the mean age of the atorvastatin group was 51 years, significantly higher than the 41 years observed in the non-atorvastatin group. Additionally, the atorvastatin group had a slightly higher percentage of females. The cohort included diverse racial groups, with non-Hispanic whites constituting the largest proportion at 61%. The mean body mass index (BMI) was 27.8, and no significant differences in sugar and caffeine intake were observed between the groups. However, alcohol consumption was lower in the atorvastatin group, while the proportion of smokers was significantly higher (55%) compared to the non-atorvastatin group (43%). Furthermore, the prevalence of hypertension and diabetes was notably higher in the atorvastatin group, at 35 and 14%, respectively, compared to 15 and 3.6%, respectively, in the non-atorvastatin group. More than half of the subjects reported engaging in moderate physical activity, although this was less prevalent in the atorvastatin group (35%). Detailed information is provided in Figure 1 and Table 3.

**Table 3:** Baseline characteristics of the study participants (*n* = 18,614).

Characteristic	*N* ^1^	Overall, *N* = 85,285,536^2^	No memory loss, *N* = 82,454,242^2^	Memory loss, *N* = 2,831,294^2^	*p*- value^3^
Age	18,614	42 ± (15)	41 ± (15)	51 ± (19)	<0.001
Gender	18,614				<0.001
Male		10,330 (58%)	9,918 (58%)	412 (50%)	
Female		8,284 (42%)	7,899 (42%)	385 (50%)	
Race	18,614				0.4
Mexican American		4,068 (12%)	3,894 (12%)	174 (11%)	
Other hispanic		1,780 (6.9%)	1,698 (6.9%)	82 (8.0%)	
Non-hispanic white		6,906 (61%)	6,590 (61%)	316 (59%)	
Non-hispanic black		3,835 (12%)	3,689 (12%)	146 (13%)	
Other race		2,025 (8.1%)	1,946 (8.1%)	79 (9.4%)	
Education level	18,614				<0.001
Below high school		5,008 (27%)	4,626 (25%)	382 (48%)	
High school		4,299 (24%)	4,102 (24%)	197 (25%)	
Above high school		9,307 (49%)	9,089 (51%)	218 (27%)	
BMI	18,614	27.8 ± (4.6)	27.8 ± (4.6)	28.1 ± (4.9)	0.3
Sugar	18,614	123 ± (84)	123 ± (84)	127 ± (100)	0.8
Caffeine	18,614	173 ± (222)	173 ± (219)	182 ± (281)	0.10
Alcohol	18,614	14 ± (35)	14 ± (35)	12 ± (34)	0.004
Smoking	18,614				<0.001
Yes		7,828 (43%)	7,418 (43%)	410 (55%)	
No		10,786 (57%)	10,399 (57%)	387 (45%)	
Hypertension	18,614				<0.001
Yes		3,252 (16%)	2,951 (15%)	301 (35%)	
No		15,362 (84%)	14,866 (85%)	496 (65%)	
Moderate activites	18,614				<0.001
Yes		8,246 (50%)	8,001 (50%)	245 (35%)	
No		10,368 (50%)	9,816 (50%)	552 (65%)	
Diabetes	18,614				<0.001
Yes		1,051 (4.0%)	911 (3.6%)	140 (14%)	
No		17,563 (96%)	16,906 (96%)	657 (86%)	
Atorvastatin	18,614				<0.001
No		16,390 (89%)	15,857 (90%)	533 (69%)	
Yes		2,224 (11%)	1,960 (10%)	264 (31%)	

^1^*N* not missing (unweighted). ^2^Mean ± (SD); *n* (unweighted) (%). ^3^Wilcoxon rank-sum test for complex survey samples; chi-squared test with Rao & Scott’s second-order correction.

Logistic regression results In this study, logistic regression models were employed to investigate the potential relationship between atorvastatin use and memory confusion. We constructed three models, progressively controlling for confounding factors to more accurately assess this relationship. Model 1 (unadjusted) compared the risk of memory confusion between atorvastatin users and non-users without accounting for any confounders. The results indicated that atorvastatin users were 30% more likely to experience memory confusion compared to non-users (OR = 1.3, 95% CI: 1.1–1.5, *p* < 0.001). However, this finding may have been influenced by unadjusted confounders. Model 2 (adjusted for demographic variables) included age, gender, race, and education to control for demographic effects. Following adjustment, the association between atorvastatin use and memory confusion changed; although it remained statistically significant (OR = 0.78, 95% CI: 0.51–1.1, *p* < 0.001), the strength and direction of the association weakened. This suggests that demographic variables moderate the relationship. Model 3 (fully adjusted) included all potential covariates. After accounting for all confounders, atorvastatin use was found to be significantly associated with a reduced risk of memory confusion (OR = 0.47, 95% CI: 0.15–0.79, *p* = 0.004). This indicates that atorvastatin may have a protective effect against memory confusion after controlling for other influential factors (Table 4).

**Table 4:** Association between taking Atorvastatin and memory loss.

[OR (95% CI) *p*- value]	Model 1	Model 2	Model 3
Atorvastatin			
0	Ref	Ref	Ref
1	1.3 (1.1,1.5) <0.001	0.78 (0.51,1.1) <0.001	0.47 (0.15,0.79) 0.004

**Figure 2:**
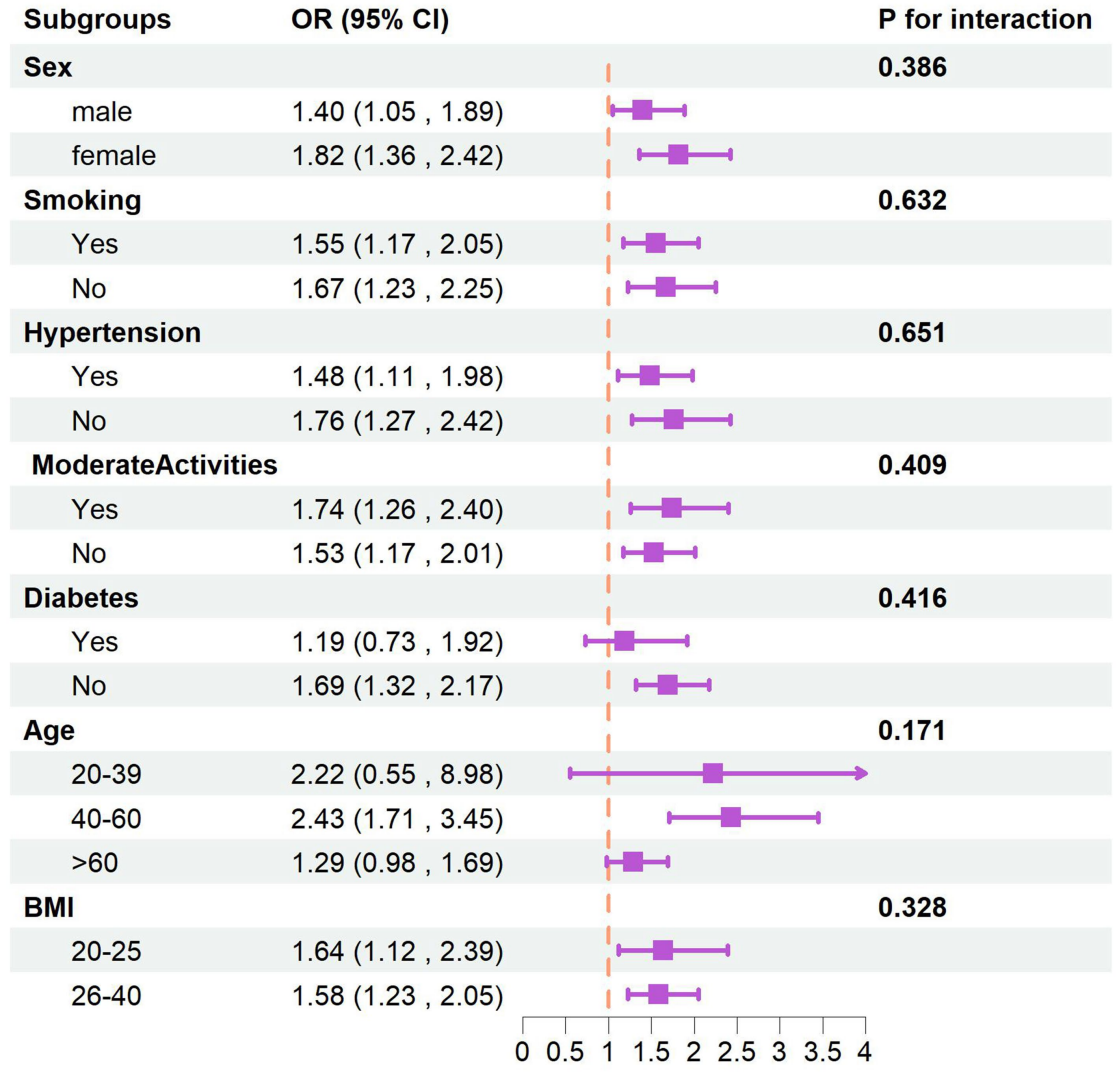
The subgroups analysis of taking atorvastatin and memory.

Interaction and Subgroup Analyses After performing stratified analyses by introducing covariates such as gender, age, BMI, smoking status, hypertension, physical activity, and diabetes, we found that the *p*-values for the interactions between these covariates and atorvastatin use with respect to memory confusion were all greater than 0.05. This indicates that these covariates did not significantly influence the primary association between atorvastatin use and memory confusion. Details of these interactions can be found in Figure 2.

**Figure 3:**
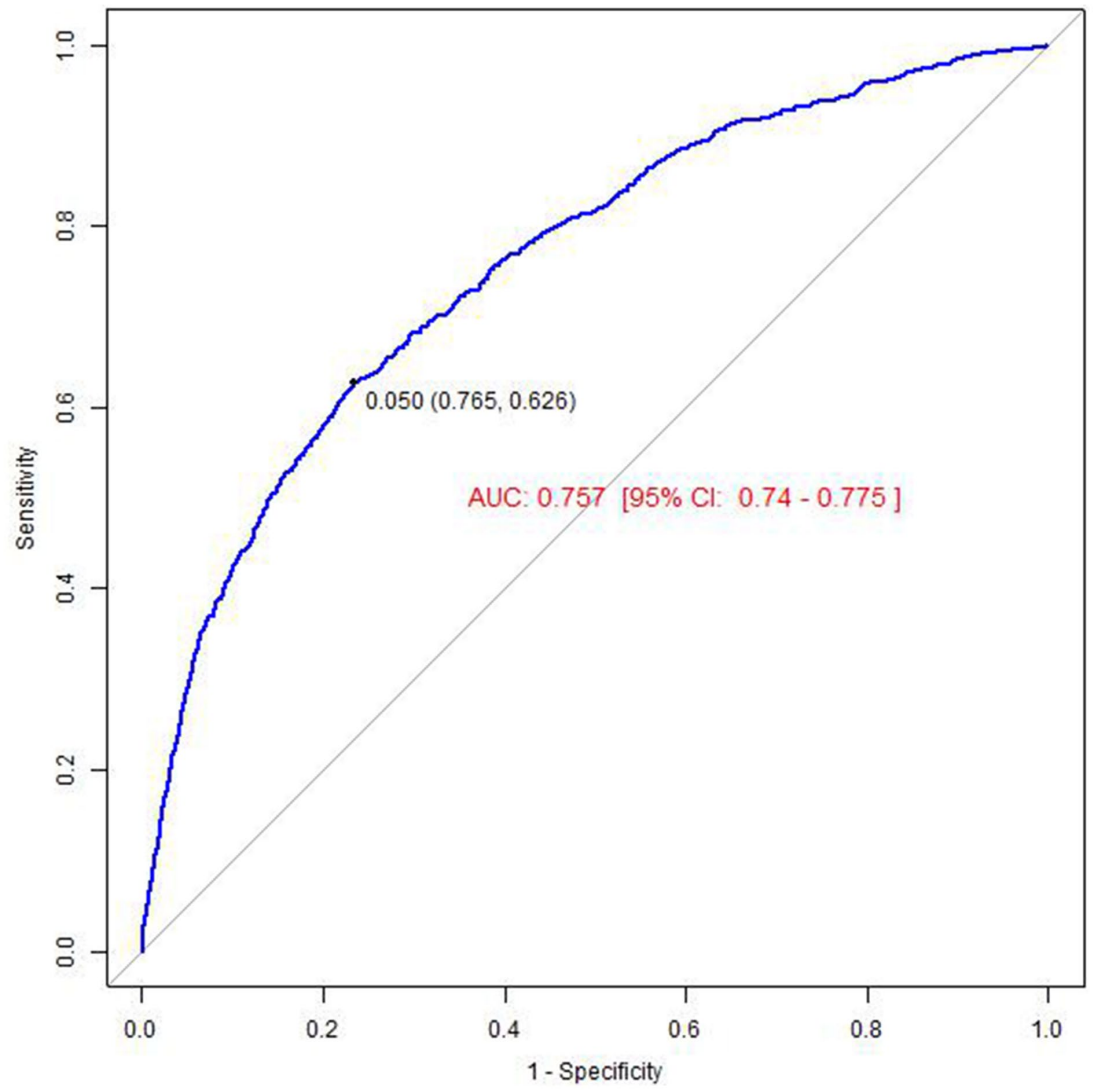
The result of receiver operator curve (ROC) (atorvastatin).

**Figure 4:**
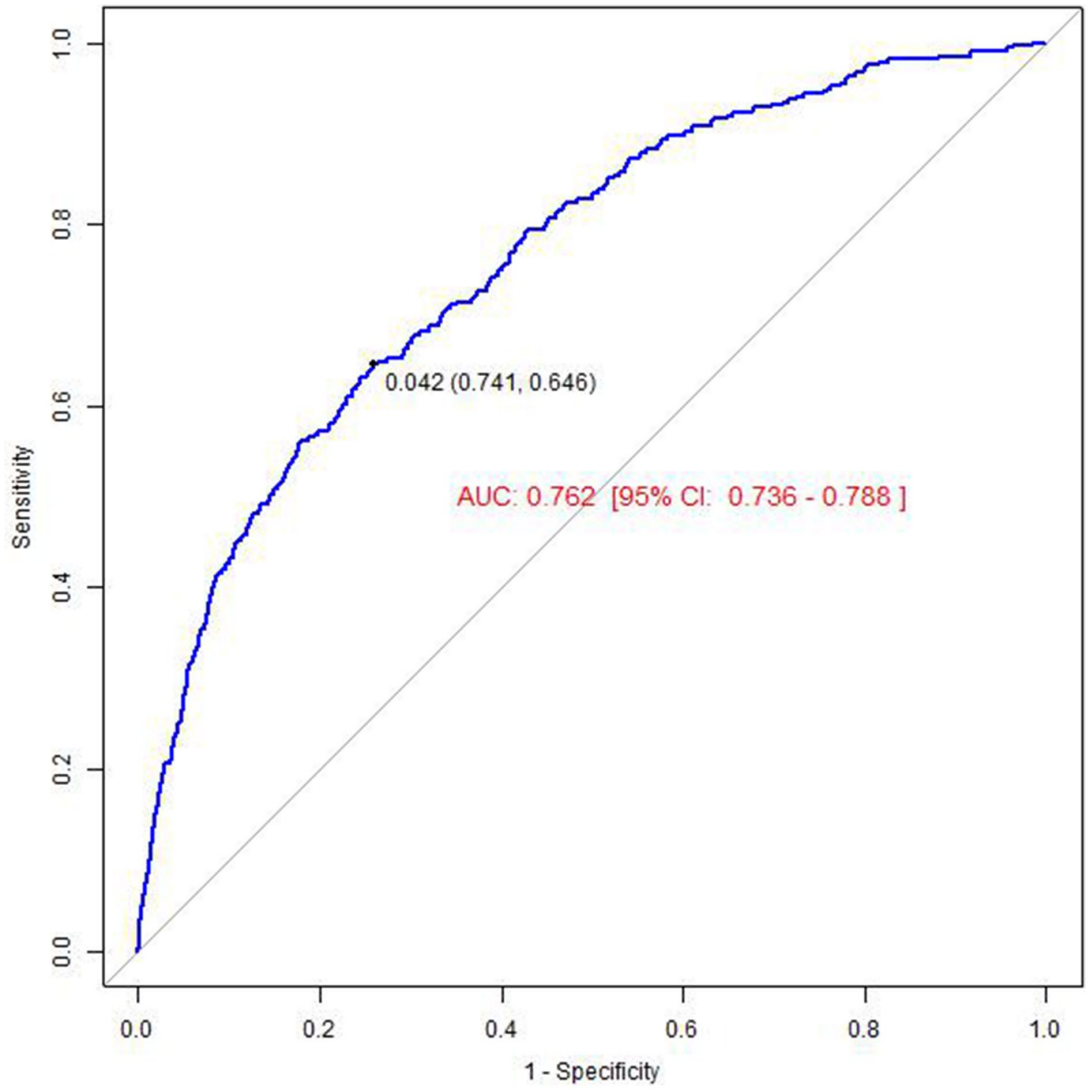
The result of receiver operator curve (ROC) (LDL-HDL).

Sensitivity analyses First, we calculated the E-Value, which was 1.60 after full adjustment for covariates. The E-Value, being above the traditional threshold, indicates that the presence of potential unobserved confounders is unlikely to entirely invalidate the observed negative association between atorvastatin use and memory confusion. Additionally, to assess the predictive ability of our model, we calculated the area under the receiver operating characteristic curve (ROC AUC). The ROC AUC value was 0.757 (95% CI: 0.740–0.775), suggesting that our model demonstrates a high degree of accuracy and reliability in predicting the risk of memory confusion, as detailed in Figure 3. The ratio of low-density lipoprotein cholesterol (LDL-C) to high-density lipoprotein cholesterol (HDL-C) is an important lipid marker, particularly significant for predicting health outcomes. However, the results revealed no significant difference in the area under the receiver operating characteristic (ROC) curve for the two: the AUC for the LDL-C/HDL-C ratio was 0.762 (95% CI: 0.736–0.788), as shown in Figure 4. This finding suggests that, within the database used in this study, both LDL-C/HDL-C ratio and individual lipid markers provide comparable predictive value for related health outcomes. This could further validate the effectiveness of atorvastatin and offer more comprehensive guidance for clinical practice. Additionally, we performed subgroup analyses, but the final logistic regression analysis indicated no particularly effective subgrouping, which may be related to the data characteristics or the population included, as detailed in Table 5.

**Table 5:** Association between taking atorvastatin and memory loss by subgroups.

[OR (95% CI) *p*- value]	Model 1	Model 2	Model 3
Hypertension			
0	Ref	Ref	Ref
1	0.93 (0.65,1.2) <0.001	0.54 (0.13,0.95) 0.010	0.48 (0.03,0.94) 0.038
Non-hypertension			
0	Ref	Ref	Ref
1	1.0 (0.69,1.4) <0.001	0.56 (0.14,0.98) 0.010	0.49 (0.01,0.97) 0.048
Diabetes			
0	Ref	Ref	Ref
1	0.39 (0.22,1.0) 0.2	0.15 (0.05,0.66) 0.7	0.21 (0.11,0.66) 0.6
Non-diabetes			
0	Ref	Ref	Ref
1	1.2 (0.97,1.4) <0.001	0.74 (0.43,1.1) <0.001	0.56 (0.22,0.90) 0.001
Age (20–39)			
0	Ref	Ref	Ref
1	0.94 (0.59,2.5) 0.2	1.0 (0.40,2.5) 0.2	0.56 (0.96,2.1) 0.5
Age (40–60)			
0	Ref	Ref	Ref
1	1.2 (0.82,1.5) <0.001	1.4 (0.99,1.7) <0.001	1.2 (0.71,1.6) <0.001
Age (>60)			
0	Ref	Ref	Ref
1	0.48 (0.19,0.78) 0.001	0.52 (0.23,0.81) <0.001	0.16 (0.08,0.53) 0.4

**Figure 5:**
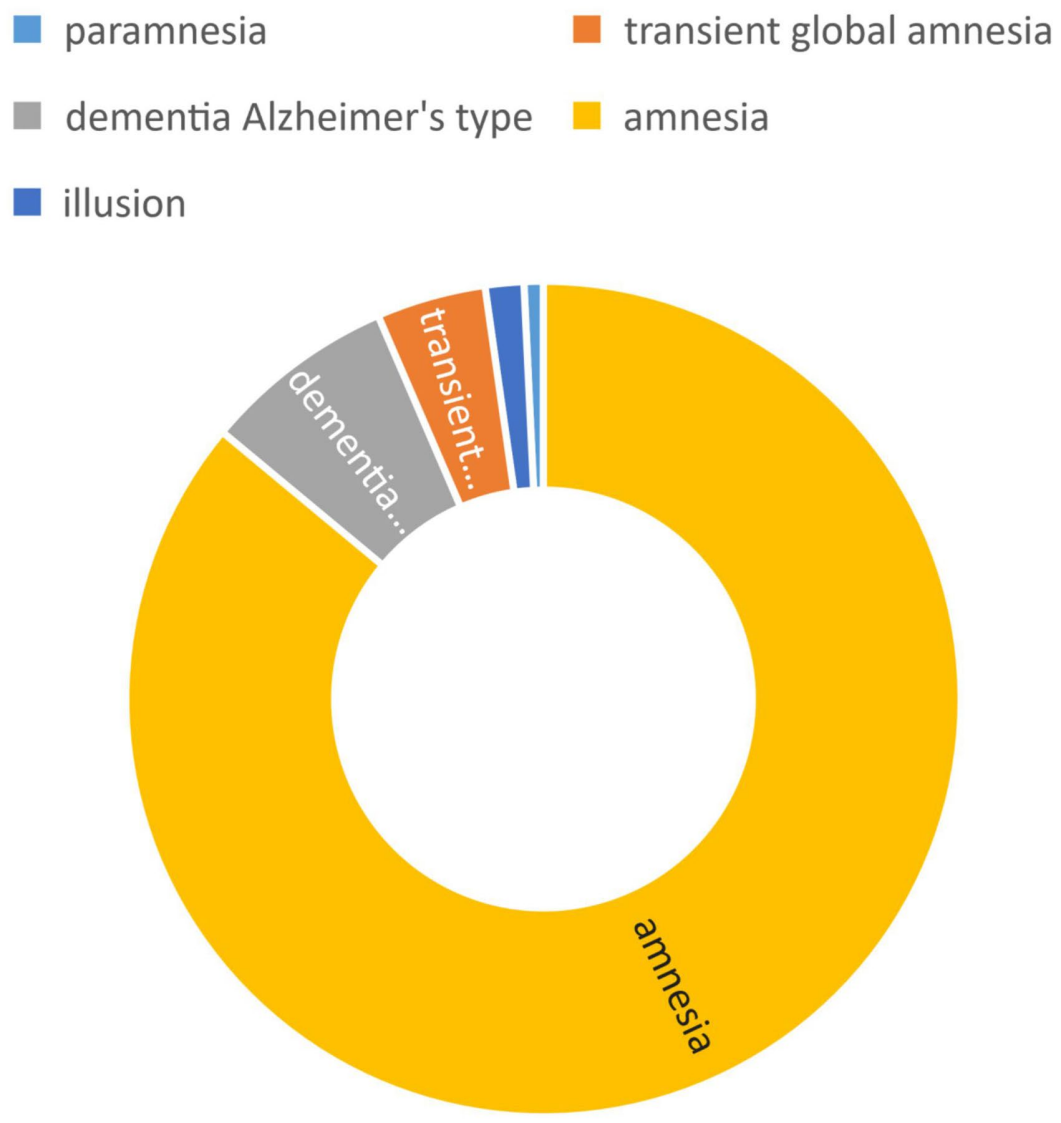
Adverse events count and composition ratio.

**Figure 6:**
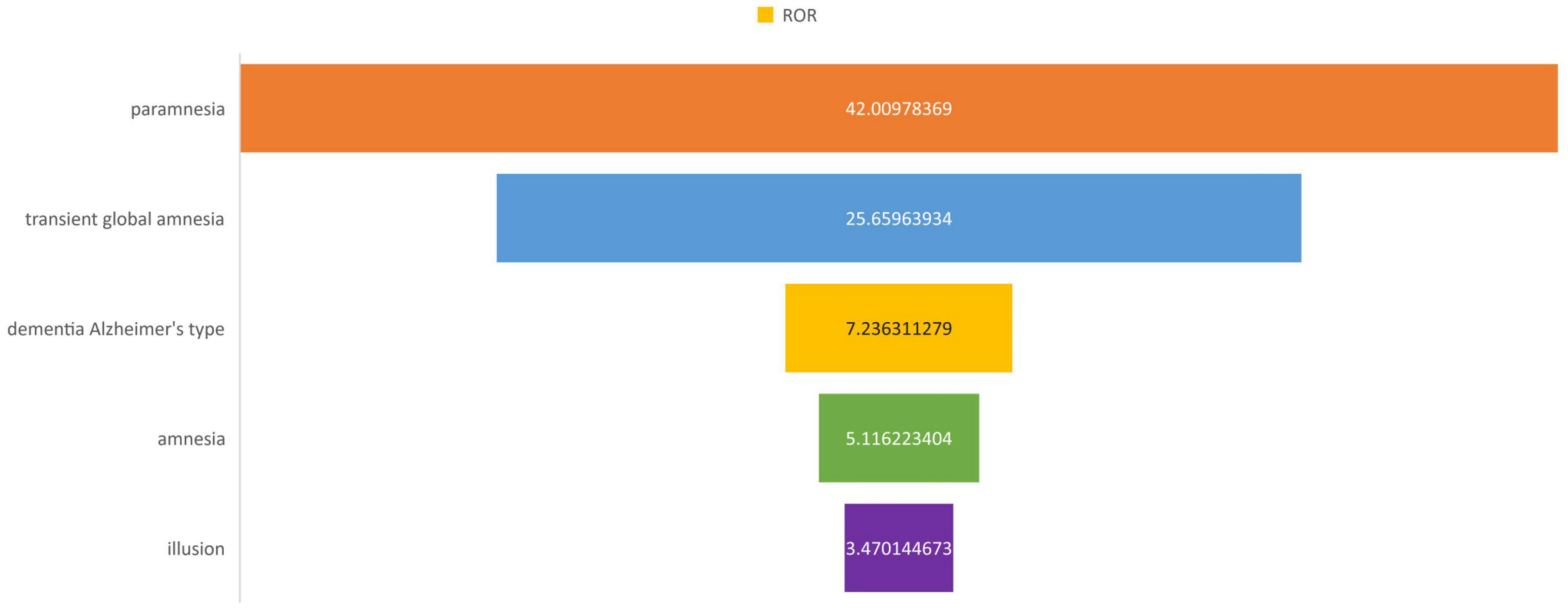
PT of signal intensity.

FAERS database results In the data mining process using the FAERS database, we identified and counted reports of adverse events associated with atorvastatin use, specifically focusing on those initially suspected to be related to this drug. For the adverse event of memory loss, we retrieved a total of 401 reports, with the distribution detailed in Figure 5. This finding suggests a potential association between atorvastatin use and memory loss. To evaluate this association more scientifically, we calculated the ratio of reported ratios (RORs) and their 95% confidence intervals (CIs), as summarized in Figure 6. The analysis revealed that for the adverse event of amnesia, the ROR value for atorvastatin was 5.12 (95% CI: 4.59–5.70), and for delusion, the ROR value was 3.47 (95% CI: 1.55–7.78), both indicating statistically significant associations.

## 4 Discussion

In the present study, we systematically utilized the extensive data resources of the National Health and Nutrition Examination Survey (NHANES) from 2001 to 2018 to explore the potential association between atorvastatin exposure and memory performance. Through rigorous statistical analyses and multivariate adjustments for various potential confounders—including demographic characteristics, dietary habits, smoking and drinking behaviors, prevalence of hypertension and diabetes, and physical activity—our results indicated that atorvastatin users had a significantly lower risk of memory distress compared to controls who did not use the medication. This association remained statistically significant even after accounting for the complex network of confounding factors, underscoring the potential benefits of atorvastatin in this context. However, interaction and subgroup analyses revealed that the *p*-values for interactions between covariates and atorvastatin use did not reach the statistical significance threshold (*p* > 0.05), suggesting limited direct moderating effects of these covariates on the relationship between atorvastatin and memory. The enhanced E-Value from sensitivity analyses further supported the robustness of our findings, indicating that unobserved confounding factors are unlikely to fundamentally undermine the validity of the results. Additionally, the receiver operating characteristic (ROC) curve analysis demonstrated excellent predictive power, validating the reliability of our analytical framework. We also screened real-world data from the FDA Adverse Event Reporting System (FAERS) for adverse reactions related to atorvastatin, identifying a number of reported memory loss events. While these cases represent a minority, they suggest a potential risk of cognitive-related side effects in specific patient populations, providing valuable insights for individualized medication decisions and patient education in clinical practice. In summary, this study enhances the understanding of the complex relationship between atorvastatin and memory and underscores the importance of drug safety and efficacy monitoring through comprehensive data analysis.

The investigation into atorvastatin’s impact on cognitive function has yielded complex findings. Gong et al. used an ApoE−/− mouse model to simulate a hypercholesterolemic environment and compared atorvastatin-treated groups with control groups. Their study suggested that atorvastatin might mitigate hypercholesterolemia-induced memory impairment by modulating PSD-95 and BDNF gene expression pathways, potentially offering protective effects (40). Additionally, Adalberto A. Castro et al. proposed that atorvastatin could alleviate memory loss in Parkinson’s disease patients by increasing nerve growth factor levels in the striatum and hippocampus and activating anti-inflammatory mechanisms (41). Li’s research on a vascular dementia model demonstrated that atorvastatin could reduce memory loss by influencing neuronal apoptosis and autophagy-related pathways, as well as activating the AMPK/mTOR signaling pathway, thereby attenuating learning and memory impairments (42). Wang’s study explored atorvastatin’s mechanism in Alzheimer’s disease, suggesting that it might help treat vascular dementia by modulating Toll-like receptor 4 (TLR4) involvement in *β*-amyloid deposition-induced neuroinflammation, inhibiting microglia and astrocyte overactivation, and reducing apoptosis, which positively impacts cognitive function (43). Conversely, Murphy et al. found no significant memory improvement with atorvastatin in an aged Beagle model, particularly with β-amyloid accumulation, and high-dose atorvastatin (80 mg/d) did not produce expected cognitive benefits (44). Similarly, Kobalava et al. observed that high-dose atorvastatin (80 mg/d) did not significantly improve cognitive function in patients with cardiovascular disease and dyslipidemia (45). A 2012 FDA warning highlighted the risk of potential memory loss associated with atorvastatin (32), with transient memory deficits reported in some patients, which resolved after discontinuation (24). These findings underscore the potential cognitive side effects of atorvastatin, with mechanisms not fully elucidated but significant enough to warrant clinical caution. Healthcare professionals should consider patients’ overall health, individual differences, and the risk–benefit profile of atorvastatin when formulating treatment plans. Future research should examine the effects of population characteristics, dosage, and duration of atorvastatin use on cognitive function to better balance cardiovascular benefits with cognitive risks and support informed clinical decision-making.

## 5 Limitations

The NHANES database, being a cross-sectional study primarily based on questionnaires, is inherently observational in nature. This design, combined with the absence of certain covariates and the reduced sample size, introduces potential biases that may impact the study’s findings. Similarly, the FAERS database, a self-reporting system, is subject to issues such as omission, misreporting, and incomplete data. The diverse sources of reporting (e.g., pharmaceutical companies, patients, physicians) further contribute to reporting bias (46). While this study employed both the ROR and BCPNN methods to enhance the ADE signal detection threshold, the possibility of false-positive ADE signals cannot be fully excluded. Consequently, this study can only establish an association and does not confirm causality. Additionally, as memory is influenced by numerous factors, future research should focus on prospective studies to further elucidate these relationships.

## 6 Conclusion

In conclusion, considering the potential memory alterations associated with atorvastatin use, it is imperative that clinicians prioritize the vigilant monitoring of cognitive function, particularly memory, during treatment. Healthcare professionals should establish comprehensive medication surveillance protocols, conducting regular assessments to detect early signs of cognitive decline. Furthermore, patients should be encouraged to adopt beneficial lifestyle modifications, including maintaining a balanced diet, engaging in regular physical activity, ensuring sufficient sleep, and managing stress—all of which may reduce the risk of memory impairment while enhancing the therapeutic efficacy of atorvastatin. In the future, we plan to engage in in-depth discussions with relevant departments, such as the neurology department, to explore how our research findings can be better applied to clinical practice. Specifically, we aim to encourage clinicians to actively inquire whether patients on long-term atorvastatin therapy exhibit symptoms such as spontaneous memory impairment, in order to identify and address potential cognitive effects at an early stage. These initiatives will help bridge the gap between the significance of the research and clinical reality, ultimately providing more comprehensive care for patients. By integrating individual variability, drug responsiveness, and lifestyle factors into personalized treatment plans, clinicians can strike an optimal balance between the cardiovascular benefits of atorvastatin and its potential cognitive risks, ultimately improving patient health outcomes.

## Data Availability

The original contributions presented in the study are included in the article/supplementary material, further inquiries can be directed to the corresponding author/s.

## Footnotes

^1^ http://openvigil.sourceforge.net/
